# Risk-taking and fairness among cocaine-dependent patients in dual diagnoses: Schizophrenia and Anti-Social Personality Disorder

**DOI:** 10.1038/s41598-020-66954-2

**Published:** 2020-06-22

**Authors:** Gerardo Sabater-Grande, Gonzalo Haro, Aurora García-Gallego, Nikolaos Georgantzís, Noemí Herranz-Zarzoso, Abel Baquero

**Affiliations:** 1 LEE & Department of Economics, Universitat Jaume I, Castellón, Spain; 2 TXP Research Group, Universidad Cardenal Herrera-CEU, CEU Universities, Valencia, Spain; 3 Amigó Foundation, Castellón, Spain; 4 Department of Mental Health, Hospital Provincial de Castellón, Castellón, Spain; 5 CEREN EA 7477, Burgundy School of Business, Université Bourgogne Franche-Comté, Dijon, France

**Keywords:** Diagnosis, Comorbidities, Health care, Signs and symptoms

## Abstract

This study reports experimental results from a clinical sample of patients with a cocaine-related disorder and dual diagnosis: Schizophrenia and Anti-Social Personality Disorder. Both types of patients as well as a non-clinical group of students performed two incentivized decision-making tasks. In the first part of the experiment, they performed a lottery-choice task in order to elicit their degree of risk aversion. In the second part, they decided in two modified dictator games aimed at eliciting their aversion to advantageous and disadvantageous inequality. It is found that the Anti-Social Personality Disorder group exhibits no significant differences from the non-clinical sample in either task. However, compared with the students’ sample, subjects from the group with schizophrenia show more risk aversion and exhibit more aversion towards disadvantageous inequality.

## Introduction

Cocaine is the most commonly used illicit stimulant drug in Europe. It is estimated that about 2.3 million young adults aged from 15 to 34 (1.9% of this age group in the general population) consumed cocaine during 2017^1^. In Spain, cocaine is responsible for the highest share of drug-related cases admitted for treatment (36.5%) and it is involved in about half of all reported drug-related emergencies^1^. Two of the most prevalent comorbid (dual pathology) mental disorders in cocaine abusers are schizophrenia and Anti-Social Personality Disorder (ASPD, henceforth)^2,3^.

Schizophrenia is a mental disorder characterized by misinterpretation in thinking processes, especially delusions (beliefs that are not based on reality) and hallucinations (inability to differentiate between what is real and what is being produced in the brain). ASPD is a personality disorder characterized by a dominant pattern of antisocial behaviors including reckless neglect of own or others’ safety, constant irresponsibility with economic obligations, and deception leading to personal gain.

The etiological studies available are aimed at unveiling causal relationships and do not actually allow for an advance in the pharmacological treatment of these two pathologies. Interestingly however, the experimental paradigm of decision-making processes in mental disorders such as addiction, psychopathy and schizophrenia^4^ has proved to be useful in the design of psychosocial treatments in patients with dual pathology.

Decision-making tasks require the activation of a complex system of cognitions, emotions, and conducts directly related to the functionality of everyday life. As^5^ states, when subjects with different personality disorders face decision-making problems, they are strongly affected by concerns related to past experiences. Situations involving risk and/or interpersonal comparisons are likely to play an important role in subjects’ decisions.

This paper focuses on the differences between, on one hand, a non-clinical sample and, on the other hand, two clinical samples involving cocaine abuse patients: one with schizophrenia and the other with ASPD comorbidities. Specifically, comparisons across the controls and the clinical samples are made to identify behavioral differences in the domains of risk and inequality aversion. Following standard terminology, risk aversion is defined as the decision maker’s disutility due to the dispersion of possible monetary consequences of uncertain prospects. Inequality aversion is a decision maker’s preference for egalitarian distributions of wealth as opposed to having *more* or *less* than others (*advantageous* and *disadvantageous* inequality aversion, respectively, as defined by^6^).

Rather than asking subjects about their preferences, *incentive-compatible* experiments face subjects with the real monetary consequences of their choices. The use of such experiments in the study of social preferences and fairness is frequently based on two related but distinct two-player paradigms: Dictator Game (DG) and Ultimatum Game (UG)^7^. In DG one of the players (dictator) decides on the shares of both players, while the other player passively experiences the consequence of this decision. In UG, the first player decision is a proposal for the second player to accept or reject, in which case they both earn nothing. While offers in the UG may also contain information on the proposer’s belief on what the second player would accept, offers in the DG are only related to the first player’s preference for fairness. The DG is adopted here as the elicitation device for inequality aversion. In fact, following^8^, a modified version of the DG allows us to elicit advantageous inequality aversion. Furthermore, we construct a symmetric task to elicit, also, disadvantageous inequality aversion. Finally, in order to simplify the task and to facilitate comparability with a larger sample, the number of alternative options available has been reduced, without any loss of information. The reason for omitting some options was the fact that, as observed in^8^, less than 5% of relevant choices were made within the range of those options. A further advantage of using this modified version of the DG is the salience (payoffs are 30–150 times higher than those in^9–11^) of the payoffs, making relevant for the subjects to carefully consider the consequences of their choices.

To the best of our knowledge,^12^ is the only previous study associating social decision-making with dual diagnoses. That paper uses the UG as a social preference elicitation device, and looks into a broader spectrum of personality disorders (antisocial, obsessive-compulsive, borderline, …). It is found that patients with dual diagnosis show poorer emotion recognition than healthy controls.

This paper fills the gap on social preference elicitation among schizophrenia or ASPD patients. We compare each one of the clinical cocaine abuse groups (schizophrenia and ASPD) with the non-clinical one.

In related literature, while no significant correlation was found between schizotypal traits and the amount of money offered in the DG, patients with schizophrenia appeared to accept more unfair offers than the controls in the UG^13,14^. Furthermore, joint analysis of UG and DG games leads to mixed results^15–18^ concerning the fairness hypothesis (that is, equality between UG and DG offers).

The association between risk aversion and personality disorders has been studied under a plethora of tasks and paradigms. Most studies have not used real monetary rewards while using some tasks from the experimental economics toolbox like the Balloon Analogue Risk Task (BART)^19–21^, the Risky-Gains Task^22^. In general, patients are more conservative, leading to suboptimal risky decisions. Contrary to this evidence,^23^ find that patients with schizophrenia reveal an overall riskier performance on the Game of Dice Task^24^.

The evidence on the relationship between psychopathy and risk aversion is mixed. All possible results have been reported. Some find that risk-aversion is increasing^25^, decreasing^26–29^ or is not related with psychopathy^30,31^.

Summing up, we aim at identifying differences between healthy controls and cocaine-dependent comorbid patients, in other-regarding (inequality aversion) and individual (risk aversion) decision-making tasks. We hypothesize that, generally speaking, schizophrenia and ASPD have effects on the subject’s attitude towards risk as well as on other-regarding feelings, not only in absolute, but also in relative terms. There is no clear pattern documented by previous research and, thus, no ex-ante clear theoretical expectation regarding the two aforementioned effects.

## Materials and methods

### Participants and procedures

Our experiment was carried out in two parts. In the first part, 220 undergraduate students participated in the experimental sessions run in the Laboratorio de Economía Experimental (LEE) of the Universitat Jaume I (UJI) in Castellón (Spain). Prior to the experimental session, informed consent was obtained from all students acknowledging acceptance of our data management protocols involving anonymity, confidentiality and exclusive use for the present scientific research. During the year 2012 (1 March to 30 June), the tool ORSEE^32^ was used to recruit subjects among undergraduate students from different degree courses (psychology, economics, business administration, law, etc.) at the U. Jaume I. At the beginning of each session, subjects were given written instructions, which were also read aloud by the organizers. Any remaining questions were answered privately. We elicited their risk preferences and their (non-) aversion to (dis)advantageous inequality with real monetary incentives. Each session lasted about half an hour and the average payoff was €15.

In the second part, from January 2016 to November 2017, a clinical sample of patients with cocaine-related disorder was recruited at the Unit of Addictive Behaviors (UCA), the Hospital Detoxification Unit (UDH) and the Severe Dual Pathology Program (PPDG), which are health care resources implemented by the Department of Health of Castellón (Spain). Informed consent was obtained from all patients in which they were informed that their medical records would be treated with strict confidentiality and would be protected in accordance with the current legislation on data protection. The confidentiality of data and participants’ identity was guaranteed. This research complied with the guidelines set out in the World Medical Association’s Declaration of Helsinki. The Ethical Committee of the Hospital Provincial of Castellón (Spain) approved this research (11/23/2015). The entire clinical sample was evaluated and classified by clinical personnel, supervised by medical researchers, in two subgroups of patients who met DSM-IV criteria for: (i) cocaine abuse patients with schizophrenia and (ii) cocaine abuse patients with ASPD.

The sociodemographic information was collected by means of an admission interview. For the clinical variables, the Dual Diagnostic Screening Interview (DDSI) was administered, validated specifically for people with substance-related disorders in order to exclude patients with diagnoses other than schizophrenia and ASPD. If negative, the Psychiatric Research Interview for Substance and Mental Disorders for DSM-IV (PRISM-IV^33^) was used to confirm a cocaine-related disorder, schizophrenia and ASPD. Moreover, for the ASPD evaluation, the specific Psychopathy List-Revised (PCL-R^34^) was clinician-administered as a complementary method. The research assessments were administered after 7–10 days of detoxification treatment, when psychopathological stabilization had been reached. We considered that patients’ capacities for concentration and attention were acceptable to face the incentivized tasks used to elicit risk and inequality aversion. In fact, before performing the tasks, patients were trained in the use of the specific software of the experiment. Furthermore, patients were asked several (pilot) questions so that the experimenters could be sure that they fully understood the instructions for the experiment. Each patient was individually assisted to ensure full understanding of the instructions and the software. Concerning the understanding ability of substance-users,^35^ find no differences in sustained attention between cocaine-users and controls in tasks which are less demanding on working memory, such as ours. Furthermore^36–38^ find that ASPD subjects generally perform as well as healthy controls in cognitive measures like planning, attention, and working memory. Previous results on sustained attention in patients with schizophrenia are mixed^39^. In that sense, although the degree to which schizophrenia patients show intact functioning on decision-making tasks is still unclear^40,41^ (using the Iowa Gambling Test to measure decision-making ability) find performance in schizophrenia patients is similar to that of healthy controls.

At the beginning of the experimental session patients were informed that they could opt out of the experiment at any time. Finally, the same software and procedures applied for the non-clinical sample were used to elicit risk aversion and aversion to (dis)advantageous inequality through incentive-compatible methods for a sample of cocaine-abusers consisting of 23 schizophrenia and 23 ASPD patients. Each session lasted about 45 minutes and the average payoff was €14. Regarding the question of how patients perceive the incentives,^42^ states that offering monetary incentives significantly improves performance on different cognitive tasks among patients with schizophrenia. We work under the assumption that this is also valid for patients with ASPD.

A possible source of bias for the observed behavior could be demand effects due to the patients’ willingness to comply with uncontrolled perceived rules and, perhaps, the actions they might feel as desirable by the doctors. In order to minimize or at least equalize any possible demand effects across the three populations, we ran the experiments in the presence of the same group of experimenters and technical support personnel.

To reduce any biases derived from inter-observer differences, all the diagnostic testing was performed by the same research psychologist, who had previously obtained the official training for the PRISM-IV interview. In order to maintain confidentiality and reduce possible “experimenter demand effects”, diagnostic tests were performed independently of the clinical interviews, and behavioral information was not transmitted to doctors.

### Measures

#### The Sabater-G.-Georgantzis lottery panel task: A measure of risk aversion

As a measure of subjects’ risk aversion, we propose a simple task for the elicitation of risk attitudes, initially used in^43^, the Sabater-Grande and Georgantzis (SGG hereafter) lottery panel task, displayed in Table 1. In this task, subjects face eight subtasks called panels 1, 2, 3, …, 8, presented in this order on a sequence of decision screens. Each panel corresponds to ten lotteries, from which subjects have to choose their preferred one. In panels 1 to 4, each lottery is defined by the probability *p* of winning a prize of €X (else nothing) at no cost. Given that a degenerate lottery of a certain (*p* = 100%) reward (€1) is included, the unfavorable options of all other lotteries (€0) can be considered as losses with respect to the €1 certain alternative. Panels 5 to 8 set the certain option to X = €0, ruling out the possibility of losses. By inspection, the farther right subjects choose, the less risk averse they are. For comparability of choices across panels, the data analysis uses the winning probability of the lottery chosen.

**Table 1:** SGG Lottery Panel Task.

**Panel 1**										
Prob.	1	0.9	0.8	0.7	0.6	0.5	0.4	0.3	0.2	0.1
€	1	1.1	1.3	1.5	1.7	2.1	2.7	3.6	5.4	10.9
**Panel 2**										
Prob.	1	0.9	0.8	0.7	0.6	0.5	0.4	0.3	0.2	0.1
€	1	1.2	1.5	1.9	2.3	3	4	5.7	9	19
**Panel 3**										
Prob.	1	0.9	0.8	0.7	0.6	0.5	0.4	0.3	0.2	0.1
€	1	1.7	2.5	3.6	5	7	10	15	25	55
**Panel 4**										
Prob.	1	0.9	0.8	0.7	0.6	0.5	0.4	0.3	0.2	0.1
€	1	2.2	3.8	5.7	8.3	12	17.5	26.7	45	100
**Panel 5**										
Prob.	1	0.9	0.8	0.7	0.6	0.5	0.4	0.3	0.2	0.1
€	0	0.1	0.3	0.5	0.7	1.1	1.7	2.6	4.4	9.9
**Panel 6**										
Prob.	1	0.9	0.8	0.7	0.6	0.5	0.4	0.3	0.2	0.1
€	0	0.2	0.5	0.9	1.3	2	3	4.7	8	18
**Panel 7**										
Prob.	1	0.9	0.8	0.7	0.6	0.5	0.4	0.3	0.2	0.1
€	0	0.7	1.5	2.6	4	6	9	14	24	54
**Panel 8**										
Prob.	1	0.9	0.8	0.7	0.6	0.5	0.4	0.3	0.2	0.1
€	0	1.2	2.8	4.7	7.3	11	16.5	25.7	44	99

After responses to this and the subsequent inequality aversion tasks are submitted, subjects earnings from the whole experiment are determined. To calculate earnings from the SGG lottery panel task, one of the eight panels is randomly picked and each subject’s preferred lottery in it is executed.

In all panels, the winning probability is varied from *p* = 0.1 to *p* = 1 in steps of 0.1. Prizes are designed so that, within a panel, the expected value of the lotteries increases linearly with the probability of not winning by a constant *t* over a fixed gain of €1 in panels 1–4, and €0 in panels 5–8. Thus, *t* is a panel-specific risk premium, which generates an increase in the expected values of the lotteries as we move from safer to riskier options within the same panel. This parameter is increased from panel 1 to 4 and from 5 to 8. Thus, intuitively, a subject should be expected to make riskier choices when moving from panel 1 to 4 and from panel 5 to 8.

#### DIA and AIA: Testing Aversion to (dis)Advantageous Inequality

We implement two modified dictator games to elicit Advantageous Inequality Aversion (AIA) and Disadvantageous Inequality Aversion (DIA). In each task, participants play both roles, dictator and recipient, but only one of them is randomly chosen to determine the subjects’ final payoffs. The underlying assumption in both tasks is that the dictator’s preferences include two components, one for own and one for other’s gains. Due to a qualitative difference between advantageous and disadvantageous inequality, two different tasks are used in order to address the relative weight of each player’s other-regarding component in each context. With the aim of not inducing a different perception of the two tasks due to format heterogeneity, we decided to modify the advantageous inequality task in^8^ to create the disadvantageous inequality one.

In the version AIA displayed in Table 2, the dictator has to decide which part of the payoff of €15 (if any) is willing to give to the partner, then achieving a more equal distribution of payoffs. More specifically, in AIA subjects are given two lists of 16 payoff vectors where the first (second) element of each payoff vector corresponds to the dictator’s (recipient’s) payoff. Observe in Table 2 that the left-hand side payoff vector contains equal payoffs for both subjects varying from (€15, €15), (€14, €14) up to (€0, €0), whereas the right-hand side payoff vector is always (€15, €0). Subjects were asked to choose at which point, if any, they preferred to switch from the left to the right payoff vector. For the sake of consistency, participants were not allowed to switch back and forth. Therefore, inconsistent preferences were avoided with a concave utility function, given that the egalitarian outcome is “cheaper” for any decision beyond the switching point (SP). Specifically, for this task a SP value of “0” indicates that the subject prefers the left-hand side payoff to the outcome (€15, €0) in all cases considered. At the other extreme, a SP of “16” denotes a situation in which the subject prefers the outcome (€15, €0) to the egalitarian payoffs in all cases considered. In intermediate positions, a SP of “1” indicates that the subject always prefers the egalitarian payoffs to (€15, €0) except when the outcome is (€0, €0). A SP of “2” denotes a preference for all the egalitarian payoffs to (€15, €0) except for outcomes (€0, €0) and (€1, €1), and so on and so forth. We use the SP ranging from 0 to 16 in order to elicit a parameter of aversion to advantageous inequality. Using the utility function of^6^, like^8^ we calculate point estimates of the subjects’ AIA parameter (β). Given that the right-hand payoff vector is (€15, €0), the AIA parameter β is calculated as:

$${\rm{\beta }}=1-\frac{SP}{15}$$

**Table 2:** Advantageous Inequality Aversion (AIA) task.

Row	My gains	Partner’s gains	My gains	Partner’s gains
1	€15	€15	€15	€0
2	€14	€14	€15	€0
3	€13	€13	€15	€0
4	€12	€12	€15	€0
5	€11	€11	€15	€0
6	€10	€10	€15	€0
7	€9	€9	€15	€0
8	€8	€8	€15	€0
9	€7	€7	€15	€0
10	€6	€6	€15	€0
11	€5	€5	€15	€0
12	€4	€4	€15	€0
13	€3	€3	€15	€0
14	€2	€2	€15	€0
15	€1	€1	€15	€0
16	€0	€0	€15	€0

The lower the SP, the higher the value of β is, and, therefore, the higher the aversion to advantageous inequality. In the data analysis, the SP is used as the advantageous inequality score.

In the version DIA, displayed in Table 3, the left-hand side of the payoff vector contains identical equal payoffs to those contained in the AIA, but the right-hand side consists always in the pair of payoffs (€0, €15). Subjects are asked to choose at which point, if any, they prefer to switch from the left to the right-hand side pair of payoffs.

**Table 3:** Disadvantageous Inequality Aversion (DIA) task.

Row	My gains	Partner’s gains	My gains	Partner’s gains
1	€15	€15	€0	€15
2	€14	€14	€0	€15
3	€13	€13	€0	€15
4	€12	€12	€0	€15
5	€11	€11	€0	€15
6	€10	€10	€0	€15
7	€9	€9	€0	€15
8	€8	€8	€0	€15
9	€7	€7	€0	€15
10	€6	€6	€0	€15
11	€5	€5	€0	€15
12	€4	€4	€0	€15
13	€3	€3	€0	€15
14	€2	€2	€0	€15
15	€1	€1	€0	€15
16	€0	€0	€0	€15

For this task, a SP value of “0” would indicate that the subject prefers the egalitarian payoffs to the outcome (€0, €15) in all cases. In the other extreme, a SP of “16” corresponds to a subject who always prefers the outcome (€0, €15) to any of the egalitarian ones. In intermediate positions, a SP of “1” indicates that the subject prefers the egalitarian payoffs to (€0, €15) in all cases except when the outcome is (€0, €0). A SP of “2” denotes a preference for all the egalitarian payoffs to (€0, €15) except for outcomes (€0, €0) and (€1, €1), and so on and so forth. It is worth mentioning that the only reason for a subject to choose the right-hand side options is a willingness to sacrifice his/her own payoff to increase the partner’s payoff, representing a subject’s kind of generosity. We use the SP ranging from 0 to 16 with the aim of calculating the parameter of aversion to disadvantageous inequality, which we call the DIA parameter (α). As in the case of parameter β for AIA, the DIA parameter is calculated as:

$${\rm{\alpha }}=1-\frac{SP}{15}$$

Observe that the lower the value of SP, the higher the level of aversion to disadvantageous inequality is, corresponding to a higher value of α. The SP is used as the disadvantageous inequality score in the data analysis.

#### Substance use and mental disorders

##### Dual diagnosis screening interview (DDSI)

The Dual Diagnosis Screening Instrument (DDSI) was designed by^44^ to screen psychiatric disorders in substance abusers in with-treatment and without-treatment seeking samples. The DDSI has shown a valid and easy-to-administer screening tool for detecting possible psychiatric comorbidities among substance abusers. Specifically, the DDSI has a high sensitivity (≥80%) for identifying lifetime depression, mania, psychosis, panic, social phobia, and specific phobia disorders, being higher than or equal to 82% for those diagnoses. A test-retest κ shows excellent agreement: range 81%–95%. Patients with a positive diagnosis other than schizophrenia or ASPD applying this screening are excluded from the study.

##### Psychiatric Research Interview for Substance and Mental Disorders version IV (PRISM-IV)

The PRISM-IV was developed by^33^ as a semi-structured clinician-administered interview with the purpose of measuring the major Axis I DSM-IV diagnoses (current and past) of alcohol, drug and psychiatric disorders. It was designed to provide clear guidelines for differentiating among the expected effects of intoxication and withdrawal, substance-induced disorders, and primary disorders. The PRISM-IV also covers two Axis II disorders: Borderline Personality Disorder and Antisocial Personality Disorder.

Although primarily designed as a research instrument, the PRISM-IV provides coverage of alcohol- and drug-related experiences and symptoms that may be relevant in identifying areas of focus for medical treatment. The interview is used to include subjects within one of two groups: (i) schizophrenia and cocaine-related disorder, and (ii) ASPD and cocaine-related disorder. PRISM-IV is also applied to describe the different substance-related disorders, as well as to confirm cocaine-related disorders.

##### PCL-R: A Psychopathy test

The PCL-R (Psychopathy Checklist-Revised) is a 20-item measure of psychopathy designed by^33^. The items are rated on a 3-point scale (0 = item does not apply, 1 = item applies somewhat, 2 = item definitely applies). The items are summed to yield total scores, ranging from 0 to 40, reflecting the degree to which an individual resembles the prototypical psychopath. A cutoff score of 17 was used to confirm the presence of at least moderate psychopathy. The two-factor PCL-R model was used in order to examine subcomponents of psychopathy: Factor 1 (F1) assesses affective-interpersonal traits, while Factor 2 (F2) measures antisocial-impulsive traits. Extensive research attests to the reliability and validity of PCL-R total and factor scores as measures of psychopathy.

### Sociodemographic composition of the samples

This section includes a description of the sociodemographic characteristics of the samples under analysis. Concerning average age, subjects in the non-clinical sample are 21.9 years old (SD = 2.9), 39.4 years old (SD = 8.5) in the schizophrenia sample, and 43 years old (SD = 5.5) in the ASPD sample. With respect to gender, the share of males is 37.7% in the non-clinical sample, 95.2% in the schizophrenia sample and 95.7% in the ASPD sample. The non-clinical sample was randomly formed by volunteers responding to a public call, according to usual recruitment experimental protocols. As for the clinical sample, it was formed by volunteers recruited among the patients under treatment available over a two-year period, leading to a gender-unbalanced composition.

Focusing on other relevant sociodemographic characteristics, 52.2% of our patients with schizophrenia and 65.2% with ASPD have a family history of addictions. Patients started consuming cocaine at an average age of 18.7 years (SD = 4.1) for the schizophrenia sample and 18.8 years (SD = 6.2) for the ASPD sample, and developed a cocaine-related disorder at the age of 20.1 years (SD = 4.5) for the schizophrenia and 20.3 (SD = 7.2) for the ASPD groups respectively. The mean score on the cocaine severity index is 7.9 (SD = 2.7) for the schizophrenia group, and 8.0 (SD = 1.6) for the ASPD.

The percentage of patients with alcohol-related disorder is 65.2% for the schizophrenia group, and 47.8% for the ASPD. Moreover, 73.9% of the schizophrenia sample and 68.2% of the ASPD, have a cannabis-related disorder. The cocaine and other substances consumption were similar among patients with schizophrenia and ASPD, except for heroin, used by 13% of patients with schizophrenia against 86.4% in the ASPD group (*X*^2^ = 24.2; p < 0.001). Furthermore, 52.2% of the schizophrenia sample and 34.8% of the ASPD has a family history of mental illness.

Important differences are found in the psychopathological assessment. Namely, patients with ASPD scored higher than patients with schizophrenia on the total PCL-R score (Mean Difference [MD] = 26.2; p < 0.001), with significant differences in all the facets and factors of the PCL. Finally, schizophrenia patients had a higher incidence of suicide attempts than the ASPD group (47.8% vs. 30.4%; *X*^2^ = 5.7; p = 0.017).

### Data analysis and results

We present our analysis by task, explaining our empirical strategy. First, for the risk task the variable used is the probability of the lottery chosen in each panel. Second, for the aversion to (dis)advantageous inequality task, the variable used is the SP between the (altruist) selfish and the egalitarian choice. For each variable, we present descriptive statistics. When reported, correlation between variables refers to the non-parametric pairwise Spearman correlation.

Normality of the corresponding distributions is tested using the Shapiro–Wilk test. When normality is rejected, a Mann-Whitney (M-W) test is used to assess the significance of differences across the non-clinical and the clinical samples. The M-W test compares mean ranks and, in the case of identically shaped distributions (checked by a Kolmogorov-Smirnov test), is a test of medians.

The clinical and non-clinical samples are demographically different. We have checked whether differences in gender composition and age are responsible for the differences in behavior. With respect to gender, random control samples of similar composition with that of the clinical group have been generated. To do this, all possible combinations of four different females (21 groups of 4) were matched with 21 groups of 84 males formed by randomly skipping one, two or three males of the non-clinical sample. This is equivalent to any random extraction of the 17.44*10^44^ 88-subject possible samples, that can be formed from the non-clinical population with an identical gender composition as that of the clinical sample. To account for possible confounding effects of age in our clinical vs non-clinical behavioral differences, we have performed OLS regression analysis whose results are reported in Table 6. The interference of age differences across clinical and non-clinical samples in the reported behavioral differences has been ruled out.

### Risk task results

#### Descriptive statistics

Table 4 presents descriptive statistics (mean and standard deviation) corresponding to the SGG lottery panel task for the three samples: non-clinical subjects, patients with schizophrenia, and patients with ASPD.

**Table 4:** Descriptive statistics and M-W tests comparisons of SGG lottery panel task.

SGG prob.	Non-clinical (N = 222)		Schizophrenia (N = 23)		ASPD (N = 23)		Comparisons non clinical vs.	
	*M*	*SD*	*M*	*SD*	*M*	*SD*	Schizophrenia (Mean ranks)	ASPD (Median)
Panel 1	0.48	0.25	0.61	0.23	0.53	0.34	2.371**	0.323
Panel 2	0.43	0.20	0.65	0.19	0.41	0.26	4.606***	−0.909
Panel 3	0.42	0.20	0.56	0.23	0.46	0.27	2.940***	0.644
Panel 4	0.40	0.21	0.58	0.22	0.41	0.27	3.346***	−0.100
Panel 5	0.54	0.28	0.62	0.22	0.43	0.31	1.045	−1.555
Panel 6	0.50	0.27	0.56	0.27	0.45	0.28	1.047	−0.868
Panel 7	0.48	0.25	0.69	0.22	0.47	0.27	3.581***	−0.033
Panel 8	0.44	0.23	0.57	0.24	0.45	0.27	2.332**	0.228

Median and Standard Deviation corresponding to probabilities (SGG prob.) chosen by subjects in each panel of the SGG lottery panel task; (last two columns) M-W test U values. Significance values: *p < 0.1; **p < 0.05; ***p < 0.01.

Whereas choices by the non-clinical and the ASPD samples are on average below 0.5 in most panels, patients with schizophrenia choose, on average, lotteries well above 0.5 in all panels. This indicates that cocaine abuse patients with schizophrenia are more risk averse than cocaine abuse patients with ASPD.

#### Statistical test comparisons

**Figure 1:**
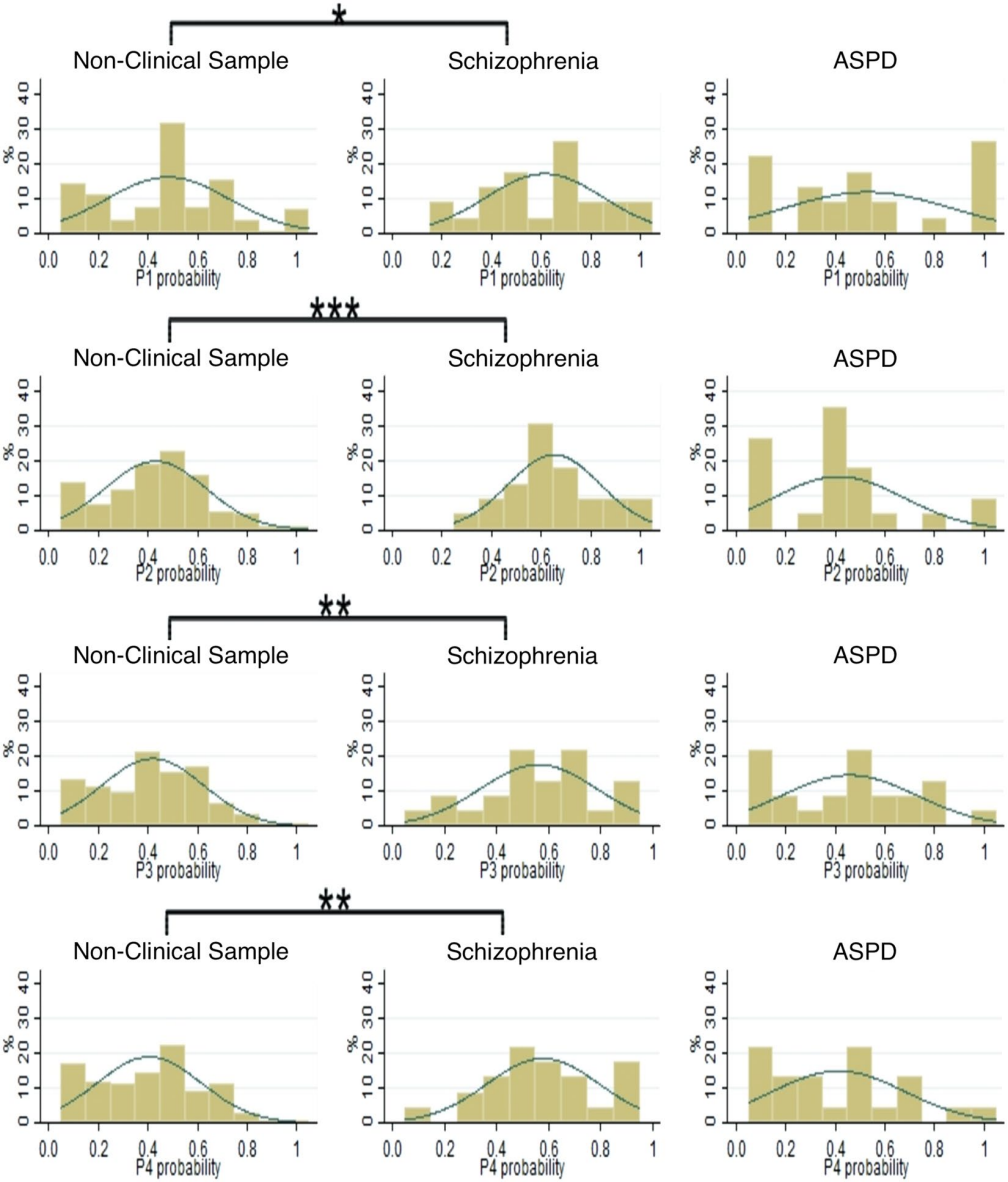
Distributions of choices in panels 1 to 4 of the SGG lottery panel task for non-clinical subjects, patients with schizophrenia and with ASPD. Kolmogorov-Smirnov significance values: *p < 0.10; **p < 0.05; ***p < 0.01.

**Figure 2:**
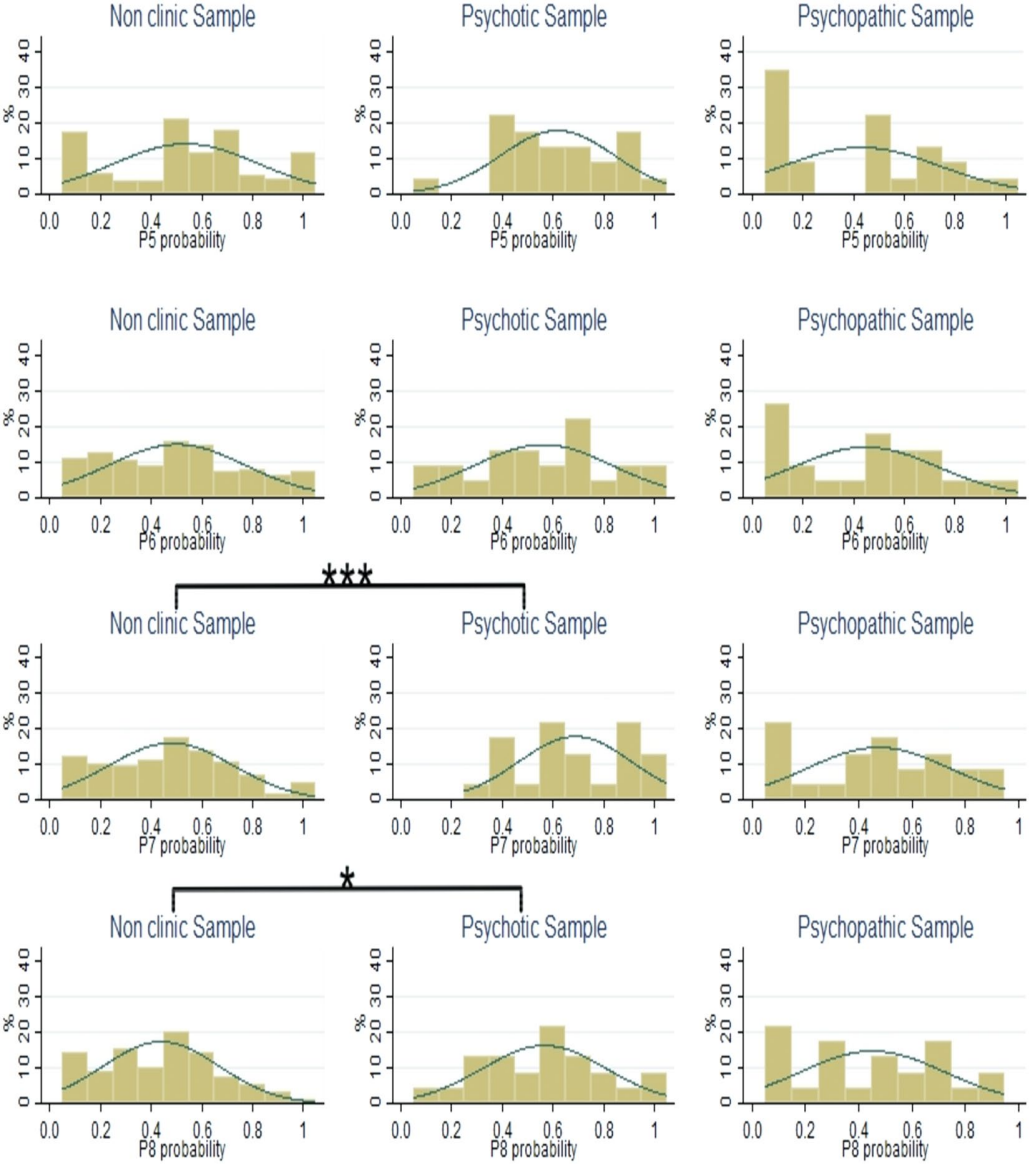
Distributions of choices in panels 5 to 8 of the SGG lottery panel task for non-clinical subjects, patients with schizophrenia and patients with ASPD. Kolmogorov-Smirnov significance values: *p < 0.10; **p < 0.05; ***p < 0.01.

The distributions of choices in the SGG lottery panel test are presented in Figs. 1 and 2. Specifically, Fig. 1 shows the distributions of choices in panels 1 to 4, and Fig. 2 presents the choices in panels 5 to 8.

The Shapiro–Wilk test rejects normality for the risk aversion data in the non-clinical sample. Figures 1 and 2 show that for four (out of the total of eight) panels in the SGG lottery panel task, there are significant differences (Kolmogorov-Smirnov test) between the distributions of choices made by cocaine abuse patients with schizophrenia and the non-clinical sample. Given that no significant differences are found between the distributions of choices made by non-clinical subjects and cocaine abuse patients with ASPD, the corresponding comparisons using the M-W test will be attributed to median risk scores.

Table 4 shows that for most panels of the SGG lottery panel task, with the exception of panels 5 and 6, the cocaine abuse patients with schizophrenia exhibit a significantly higher risk aversion mean rank than the non-clinical sample. This implies less risky behavior by the patients with schizophrenia, independently of the risk premium offered. However, when comparing the ASPD patients’ median risk aversion with the non-clinical sample, no significant differences are found.

Throughout the sample, no significant correlation (Spearman) is obtained between risk and social preferences elicitation tasks. Thus, we can proceed with the analysis of social preferences independently of risk preferences.

### AIA and DIA tasks results

#### Descriptive statistics

Table 5 shows descriptive statistics (mean SP, and standard deviation) corresponding to the AIA and DIA tasks for non-clinical subjects, patients with schizophrenia, and patients with ASPD.

**Table 5:** Descriptive statistics and M-W tests comparisons of the AIA and DIA tasks.

	Non-clinical (N = 222)		Schizophrenia (N = 23)		ASPD (N = 23)		Mean rank comparisons non clinical vs.	
SP	*M*	*SD*	*M*	*SD*	*M*	*SD*	Schizophrenia	ASPD
AIA	10.30	3.78	10.04	4.83	10.74	5.46	0.213	−0.938
DIA	11.31	4.92	6.39	6.29	9.00	6.42	0.001**	−0.962

Median and Standard Deviation corresponding to the switching point (SP) of subjects in the AIA (Advantageous Inequality Aversion) and the DIA (Disadvantageous Inequality Aversion) tasks. ASPD: Anti-Social Personality Disorder; (columns 7–8) Mann-Whitney test U values. Significance values: *p < 0.1; **p < 0.05; ***p < 0.01.

Data from the AIA test yield SP means within the (10, 11) interval for all groups, clinical and non-clinical. This evinces that subjects are willing to sacrifice an average amount between €5 and €6 of their €15 payoff, in order to reach an outcome in which the partner earns between €9 and €10.

For the non-clinical and the ASPD samples, data from the DIA test yield similar mean SP as the AIA test. Observe that, on average, in order to keep egalitarian earnings of €9, non-clinical subjects are willing to reduce the partner’s payoff by €6. However, cocaine abuse patients with schizophrenia are willing to reduce the partner’s payoff between €8 and €9 in order to reach an egalitarian outcome oscillating between €6 and €7. Therefore, on average, the exchange rate between the other’s and one’s own payoff for cocaine abuse patients with schizophrenia almost doubles the corresponding exchange rate of the other two samples, the non-clinical and the cocaine abuse patients with ASPD.

#### Statistical test comparisons

**Figure 3:**
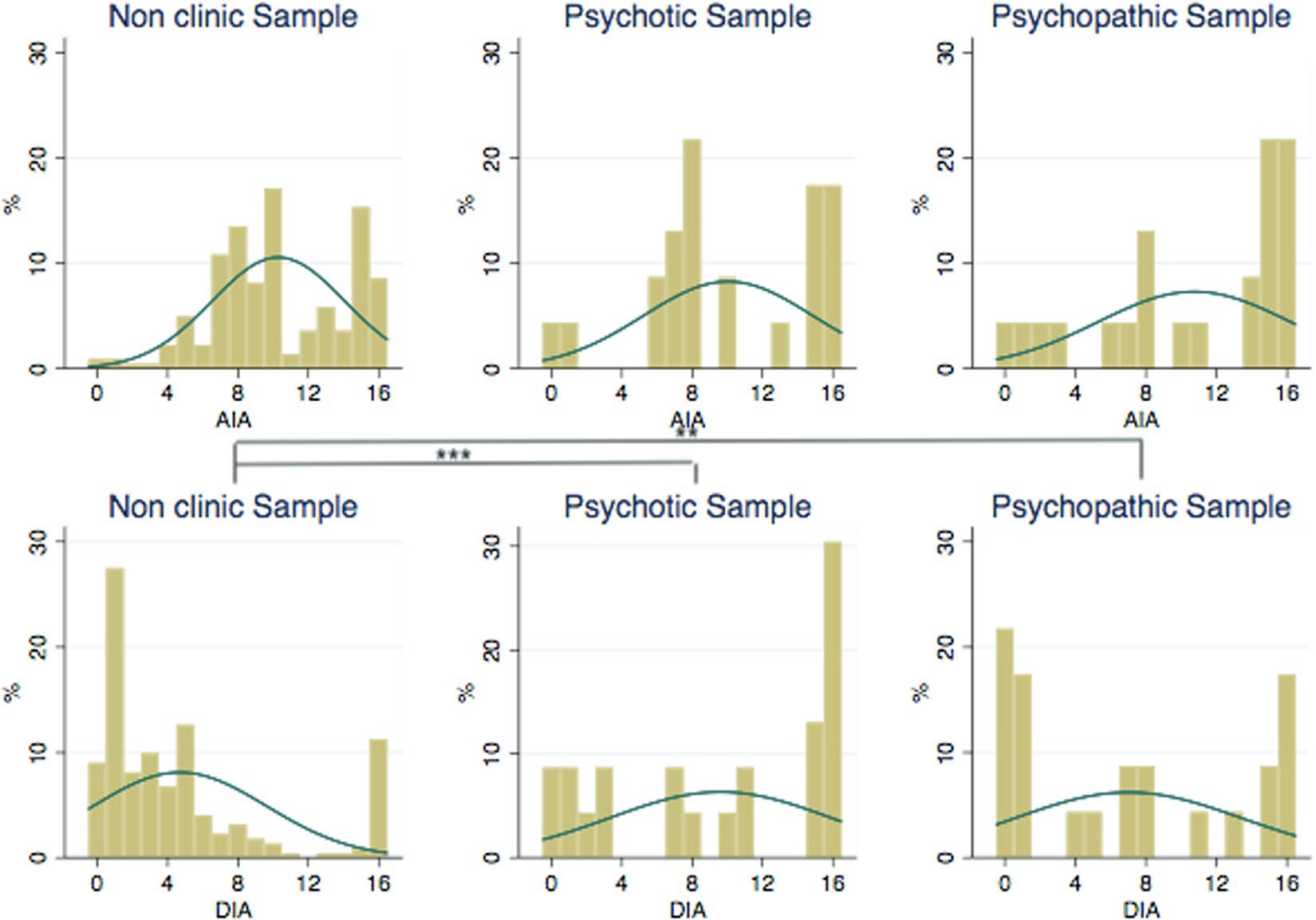
Choice distributions in AIA and DIA for non-clinical subjects, patients with schizophrenia and patients with ASPD. Kolmogorov-Smirnov test significance values: *p < 0.1; **p < 0.05; ***p < 0.01.

Figure 3 displays the distributions of choices corresponding to AIA and DIA. Like in the risk task, the Shapiro–Wilk test shows that choices in these two tests are not normally distributed for the non-clinical sample. Significant differences are obtained between the distributions of DIA of cocaine abuse patients with schizophrenia and the non-clinical subjects.

Observe in Table 5 that cocaine abuse patients with schizophrenia exhibit a significantly higher DIA mean rank than the non-clinical sample. However, no significant differences are found for DIA between cocaine abuse patients with ASPD and the non-clinical sample. Regarding AIA scores, no statistically significant differences are found between healthy controls and the clinical sample.

The present study has potential limitations concerning the sample size, possible confounding factors (subjects’ IQ, years of education, etc.), and the control group (young students). Regarding the relatively small sample size, and in order to check whether some the lack of significance for some differences was due to a low statistical power, we conduct an ex-post power analysis using Stata with power set at 0.80 and probability at 0.05. In our case, sample sizes for ASPD subjects and patients with schizophrenia would have to increase to at least N = 74 and 107, respectively, in order for group differences to reach statistical significance at the 0.05 level. As far as the control group is concerned, significant divergence in gender and age exists between the control group and the clinical sample which might be responsible for some of the differences reported. To control for such a bias attributable to gender, we have generated random non-clinical samples with the same gender proportion as the clinical one. The analysis performed based on these samples supports the robustness of the results. With respect to age discrepancies between clinical samples and controls, the robustness check has been twofold. First, using Spearman correlation, we find that age is not significantly correlated with the choices in the tasks. Second, in Table 6 we present regression analysis in which age has been used as an explanatory variable, obtaining non-significant effects on either risk or social preferences. With respect to risk, similar results have been obtained for young and midlife adults^45^ and for a larger random sample by^46^ who adopt the same risk task. With respect to social preferences, an insignificant age effect was also reported by^47,48^.

**Table 6:** Regression analysis: testing for age effects.

	Average probability chosen (Av. individual risk-taking)	AIA	AIA	DIA
ASPD	−0.043	−2.468	−6.456	2.443
	(0.061)	(1.591)	(7.114)	(2.077)
Schizophrenia	0.115**	−2.712*	−4.180	5.025***
	(0.055)	(1.426)	(4.589)	(1.860)
Age	0.002	0.138**	0.099	−0.008
	(0.002)	(0.063)	(0.094)	(0.082)
Age*ASPD			0.112	
			(0.183)	
Age*Schizophrenia			0.0545	
			(0.138)	
Constant	0.429***	7.255***	8.111***	4.903***
	(0.054)	(1.398)	(2.066)	(1.825)
Observations	262	262	262	262
R-squared	0.068	0.020	0.022	0.074

OLS Regression coefficients: risk-taking, AIA and DIA models. Explanatory variables: ASPD, Schizophrenia, age, and interaction effects. Standard errors in parentheses.

Significance values: ***p < 0.01, **p < 0.05, *p < 0.1.

Supplementary material.

Data in Brief.

Instructions to experimental subjects.

A plethora of other uncontrolled factors may partially be responsible for some of the differences reported here. Thus, our results have to be taken with caution when establishing causal relationships between the underlying pathologies and behavioral patterns of this study. Further research is needed to identify the direct or moderating effects of other socioeconomic factors. Given the difficulty in recruiting patient samples and performing behavioral experiments under the desirable conditions, the task of identifying all possible causal relationships is far from an easy one.

## Discussion

Cocaine consumers initially use the drug as an escape from personal and professional troubles^49^, thus, as a means of mood self-regulation. However, cocaine dependence is often (about 50% of cases) accompanied by mental health disorders^50^, schizophrenia and ASPD being the two most commonly co-occurring. We have hypothesized that, among the consequences of these comorbidities, non-transitory effects may be observed on risk-taking behavior and social preferences.

We address this hypothesis with data obtained in an incentivized economic experiment. Contrary to previous research^51^, no relation is found between the two domains of behavior studied here: risk and social preferences. Following standard economic theory, it is expected that risk and social preferences elicitation tasks to be independent. Thus, the relationship reported by^51^ must be attributed to the gamble-choice task^52^. To the best of our knowledge, this is the first study adopting a standard, incentivized lottery choice task, in order to elicit attitudes toward risk in clinical subjects with a dual diagnosis of either schizophrenia or ASPD. Hence, it is also the first to report, in an incentivized context, higher degrees of risk aversion associated with schizophrenia, but not with ASPD. The lack of ASPD effects on risk attitudes is in line with^53^.

Regarding the behavior domain of social preferences, it is found that they are also affected by schizophrenia. Specifically, we find that cocaine abuse patients with schizophrenia exhibit a higher aversion to disadvantageous inequality compared to the non-clinical sample.

Our results contrast with previous literature that reports either the contrary effect (^10,11^ with, and^8,54^ without monetary incentives), or no effect at all (^13,55^, using UG). This divergence with previous results may be due to differences in the elicitation method. However, it is also true that the perceived persecution and suspicious hypervigilance in schizophrenia^56,57^ may lead to an increased sensitivity towards unfavorable inequality.

### Concluding remarks

This study reports results on an incentivized experiment designed to elicit risk and social preferences of patients with a cocaine-related disorder and dual diagnosis (Schizophrenia and ASPD) and a non-clinical sample. The ASPD group exhibits no significant differences from the non-clinical sample, whereas the group with schizophrenia shows a higher aversion to risk and to disadvantageous inequality.

Our study offers a unique opportunity to compare healthy controls with cocaine abuse ASPD patients, as well as with cocaine abuse patients with schizophrenia. In terms of clinical treatment, the two groups are fundamentally different, since the former cannot be automatically considered to suffer from a treatable disease, while the other can. Furthermore, the relevance in knowing more about the risk and inequality aversion of cocaine abuse patients with schizophrenia may directly affect the mental health professionals' evaluation when facing judicial applications for the legal incapacitation of those patients to freely manage their money and to own property in general. On the contrary, cocaine abuse ASPD patients are not subject to such evaluations, since their competence in such duties is not under scrutiny by the law.

The differences reported here add evidence in favor of the differential therapeutic attitude adopted so far. An antisocial person may show levels of risk and inequality aversion similar to healthy individuals, whereas patients with schizophrenia are different from non-clinical subjects in both risk and aversion to disadvantageous inequality. Our approach also serves as proof of the potential added-value of behavioral and experimental economics in the study of different psychopathologies.

While we are still far from providing mental health professionals with a definite and reliable tool for differential diagnoses, our study shows that behavioral economics can be added as a valuable instrument to the complex toolbox of mental health professionals. More studies like ours will be needed before we can reach a full map with clear borders between different mental conditions.

## Supplementary information

Supplementary Information.

Supplementary Information 2.
