# Supplementary material for: Risk-taking and fairness among cocaine-dependent patients in dual diagnoses: Schizophrenia and Anti-Social Personality Disorder
Source: Sci Rep. 2020 Jun 22;10:10120. doi: 10.1038/s41598-020-66954-2 (PMC7308379; doi:10.1038/s41598-020-66954-2)
Supplement: Supplementary file 2 — Supplementary Information 2. [file 41598_2020_66954_MOESM2_ESM.docx]

Title: **Risk-taking and fairness among cocaine-dependent patients in dual diagnoses: Schizophrenia and Anti-Social Personality Disorder**

Authors: Gerardo Sabater-Grande, Gonzalo Haro, Aurora García-Gallego, Nikolaos Georgantzís, Noemí Herranz-Zarzoso and Abel Baquero

**Appendix. Experimental Instructions**

Welcome and thank you for participating in this experimental session. By following the instructions, you will earn an amount in euros that will be paid in cash at the end of the session. Decisions and earnings of each participant will remain anonymous throughout the session. If you have a question or problem at any point in this experiment, please raise your hand and one of the assistants will attend your request.

In this session, you will participate in two different tasks, but only one of them will be randomly selected to determine your final earnings. In what follows we give you the instructions for Task 1. You will receive the instructions for Task 2 at the end of Task 1.

**Instructions for Task 1**

The following figure will appear in your computer screen, showing eight panels of ten lotteries each.

| Panel 1 | | | | | | | | | | |
| --- | --- | --- | --- | --- | --- | --- | --- | --- | --- | --- |
| Prob. | 1 | 0.9 | 0.8 | 0.7 | 0.6 | 0.5 | 0.4 | 0.3 | 0.2 | 0.1 |
| € | 1.00 | 1.10 | 1.30 | 1.50 | 1.70 | 2.10 | 2.70 | 3.60 | 5.40 | 10.90 |
| Panel 2 | | | | | | | | | | |
| Prob. | 1 | 0.9 | 0.8 | 0.7 | 0.6 | 0.5 | 0.4 | 0.3 | 0.2 | 0.1 |
| € | 1.00 | 1.20 | 1.50 | 1.90 | 2.30 | 3.00 | 4.00 | 5.70 | 9.00 | 19.00 |
| Panel 3 | | | | | | | | | | |
| Prob. | 1 | 0.9 | 0.8 | 0.7 | 0.6 | 0.5 | 0.4 | 0.3 | 0.2 | 0.1 |
| € | 1.00 | 1.70 | 5.50 | 3.60 | 5.00 | 7.00 | 10.00 | 15.00 | 25.00 | 55.00 |
| Panel 4 | | | | | | | | | | |
| Prob. | 1 | 0.9 | 0.8 | 0.7 | 0.6 | 0.5 | 0.4 | 0.3 | 0.2 | 0.1 |
| € | 1.00 | 2.20 | 3.80 | 5.70 | 8.30 | 12.00 | 17.50 | 26.70 | 45.00 | 100.00 |

| Panel 5 | | | | | | | | | | |
| --- | --- | --- | --- | --- | --- | --- | --- | --- | --- | --- |
| Prob. | 1 | 0.9 | 0.8 | 0.7 | 0.6 | 0.5 | 0.4 | 0.3 | 0.2 | 0.1 |
| € | 0.00 | 0.10 | 0.30 | 0.50 | 0.70 | 1.10 | 1.70 | 2.60 | 4.40 | 9.90 |
| Panel 6 | | | | | | | | | | |
| Prob. | 1 | 0.9 | 0.8 | 0.7 | 0.6 | 0.5 | 0.4 | 0.3 | 0.2 | 0.1 |
| € | 0.00 | 0.20 | 0.50 | 0.90 | 1.30 | 2.00 | 3.00 | 4.70 | 8.00 | 18.00 |
| Panel 7 | | | | | | | | | | |
| Prob. | 1 | 0.9 | 0.8 | 0.7 | 0.6 | 0.5 | 0.4 | 0.3 | 0.2 | 0.1 |
| € | 0.00 | 0.70 | 1.50 | 2.60 | 4.00 | 6.00 | 9.00 | 14.00 | 24.00 | 54.00 |
| Panel 8 | | | | | | | | | | |
| Prob. | 1 | 0.9 | 0.8 | 0.7 | 0.6 | 0.5 | 0.4 | 0.3 | 0.2 | 0.1 |
| € | 0.00 | 1.20 | 2.80 | 4.70 | 7.30 | 11.00 | 16.50 | 25.70 | 44.00 | 99.00 |

Given one panel, each column indicates a lottery: a probability of earning an amount of money in €; alternatively, nothing. All lotteries have the same structure. The lowest prize is 0 euros for each lottery, while the highest prize is a positive amount of euros, this amount being different for each lottery. For each lottery, the computer screen shows the probability of winning a positive amount of euros, being zero the alternative.

For each of the eight panels of lotteries below, please indicate the lottery you would like to play. Each panel is independent of the other panels.

At the end of the experimental session, if this task is selected for payment, your earnings will be determined as follows:

•The computer randomly selects, with equal probability, one panel from the eight panels of lotteries.

•Given the panel selected and your choice in that panel, a 10-face dice is used to determine your prize.

**Instructions for Task 2**

This task has two sections (section 1 and section 2), but only one of them will be randomly selected by the computer and used for your final payment in case this is the task randomly chosen by the computer for the final payoff. In both sections the situation is as follows:

You are asked to choose between two possible distributions of money between you and your (anonymous) partner in sixteen different situations. Your partner knows that you have been asked to make such decisions, and that he has no choice but accepting.

**Section 1.** You must choose one distribution (Left or Right) in each of the 16 rows that will appear in your computer screen. If this section is chosen as the payoff relevant one the computer will randomly choose one of the sixteen decisions and the outcome in your choice will then determine your earnings.

| **Row** | **My gains** | **Partner’s gains** | **My gains** | **Partner’s gains** |
| --- | --- | --- | --- | --- |
| 1 | €15 | €15 | €15 | €0 |
| 2 | €14 | €14 | €15 | €0 |
| 3 | €13 | €13 | €15 | €0 |
| 4 | €12 | €12 | €15 | €0 |
| 5 | €11 | €11 | €15 | €0 |
| 6 | €10 | €10 | €15 | €0 |
| 7 | €9 | €9 | €15 | €0 |
| 8 | €8 | €8 | €15 | €0 |
| 9 | €7 | €7 | €15 | €0 |
| 10 | €6 | €6 | €15 | €0 |
| 11 | €5 | €5 | €15 | €0 |
| 12 | €4 | €4 | €15 | €0 |
| 13 | €3 | €3 | €15 | €0 |
| 14 | €2 | €2 | €15 | €0 |
| 15 | €1 | €1 | €15 | €0 |
| 16 | €0 | €0 | €15 | €0 |

**Section 2.** You must choose one distribution (Left or Right) in each of the 16 rows that will appear in your computer screen. If this section is chosen as the payoff relevant one the computer will randomly choose one of the sixteen decisions and the outcome in your choice will then determine your earnings.

| **Row** | **My gains** | **Partner’s gains** | **My gains** | **Partner’s gains** |
| --- | --- | --- | --- | --- |
| 1 | €15 | €15 | €0 | €15 |
| 2 | €14 | €14 | €0 | €15 |
| 3 | €13 | €13 | €0 | €15 |
| 4 | €12 | €12 | €0 | €15 |
| 5 | €11 | €11 | €0 | €15 |
| 6 | €10 | €10 | €0 | €15 |
| 7 | €9 | €9 | €0 | €15 |
| 8 | €8 | €8 | €0 | €15 |
| 9 | €7 | €7 | €0 | €15 |
| 10 | €6 | €6 | €0 | €15 |
| 11 | €5 | €5 | €0 | €15 |
| 12 | €4 | €4 | €0 | €15 |
| 13 | €3 | €3 | €0 | €15 |
| 14 | €2 | €2 | €0 | €15 |
| 15 | €1 | €1 | €0 | €15 |
| 16 | €0 | €0 | €0 | €15 |
